# Metabolic Pathways of the Warburg Effect in Health and Disease: Perspectives of Choice, Chain or Chance

**DOI:** 10.3390/ijms18122755

**Published:** 2017-12-19

**Authors:** Jorge S. Burns, Gina Manda

**Affiliations:** 1 Advanced Polymer Materials Group, University Politehnica of Bucharest, Gh Polizu 1-7, 011061 Bucharest, Romania; 2 Department of Medical and Surgical Sciences for Children & Adults, University Hospital of Modena and Reggio Emilia, 41121 Modena, Italy; 3 “Victor Babes”, National Institute of Pathology, 050096 Bucharest, Romania; gina.manda@gmail.com

**Keywords:** Warburg effect, mitochondria, radiation, metabolism, neurodegenerative diseases, space biology

## Abstract

Focus on the Warburg effect, initially descriptive of increased glycolysis in cancer cells, has served to illuminate mitochondrial function in many other pathologies. This review explores our current understanding of the Warburg effect’s role in cancer, diabetes and ageing. We highlight how it can be regulated through a chain of oncogenic events, as a chosen response to impaired glucose metabolism or by chance acquisition of genetic changes associated with ageing. Such chain, choice or chance perspectives can be extended to help understand neurodegeneration, such as Alzheimer’s disease, providing clues with scope for therapeutic intervention. It is anticipated that exploration of Warburg effect pathways in extreme conditions, such as deep space, will provide further insights crucial for comprehending complex metabolic diseases, a frontier for medicine that remains equally significant for humanity in space and on earth.

## 1. Introduction: What Is the Warburg Effect?

With the successful delivery of Mars Exploration Rovers (MER); Sojourner, Spirit (MER-A), Opportunity (MER-B) and Curiosity, plans for sending humans to Mars are in progress [1]. Beyond the considerable engineering challenges, pioneering habitation in hostile environments will require full awareness of the biological impact of galactic cosmic radiation. Like narratives describing the fate of fictional invaders from Mars [2], the final outcome will also crucially depend on a comprehensive understanding and appreciation of the microbial world. Endo-symbiotically intertwined in the evolutionary tree of eukaryotic life, mitochondria determine our overall metabolism [3]. In mammalian cells, though not all species, these ubiquitous and diverse organelles use oxygen (O_2_) as the terminal acceptor for anabolic processes, specializing our life to a dependency on oxic environments. Careful quantitative measurement of mitochondrial energy production mechanisms led to a hypothesis persisting for nearly a century as insightful for cellular stress responses to harsh environments and illness. 

**Figure 1:**
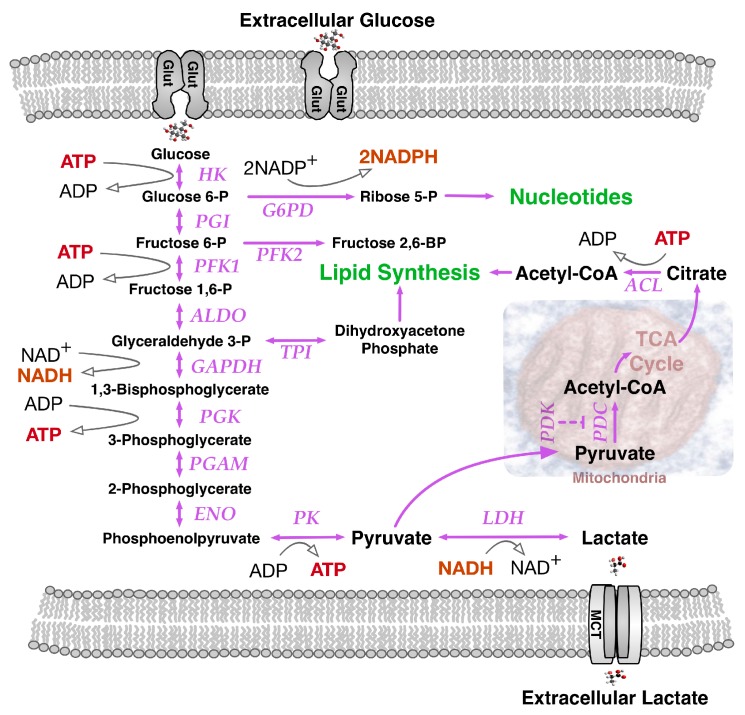
Glucose metabolism in cells. Extracellular glucose enters the cell via GLUT, a glucose transporter protein, that facilitates transport through the lipid bilayer plasma membrane. Subsequently, glucose is metabolized by pathway enzymes including HK, hexokinase; G6PD, glucose-6-phospahate dehydrogenase; PGI, phosphoglucose isomerase; PFK, phosphofructokinase; ALDO, aldolase; TPI, triose phosphate isomerase; GAPDH, glyceraldehyde 3-phosphate dehydrogenase; PGK, phosphoglycerate kinase; PGAM, phosphoglycerate mutase; ENO, enolase; PK, pyruvate kinase. Mitochondrial metabolism regulatory enzyme PDK, Pyruvate dehydrogenase kinase, phosphorylates and inactivates pyruvate dehydrogenase, the first component of PDC, the pyruvate dehydrogenase complex converting Pyruvate to Acetyl-CoA. Oxidation of Acetyl-CoA by TCA, Tricarboxylic acid cycle chemical reactions in the matrix of the mitochondria releases stored energy. The TCA metabolite citrate can be exported outside mitochondria to be broken down into oxaloacetate and acetyl-CoA by the enzyme ACL, ATP citrate lyase. Cytosolic acetyl-CoA serves as a central intermediate in lipid metabolism. When oxygen is in short supply, LDH, lactate dehydrogenase converts pyruvate, the final product of glycolysis, to lactate. MCT, monocarboxylate transporter proteins allow lactate to traverse cell membranes. Solid purple arrows, metabolite transition pathways; thin solid black arrow, coenzyme transition; Dotted T-bar, inhibition.

Dramatic changes in infant growth rate accompany the first breath of the newborn. The oxygen we breathe fuels the high energy-yielding oxyhydrogen gas reaction by which two molecules of hydrogen and one molecule of oxygen are converted to water, releasing −193 kJ/mol of energy under physiological conditions to fuel ATP synthesis from ADP and phosphate. An accompanying major source of cellular energy and new cell mass is glucose. Glycolysis breaks glucose down to pyruvate (Figure 1), that ultimately can be metabolized oxidatively to CO_2_ by a network of enzymes known as the tricarboxylic acid (TCA) cycle. Linking the metabolic pathways of glycolysis and the TCA cycle, a pyruvate dehydrogenase complex (PDC) made of three enzymatic proteins in the mitochondrial matrix breaks down pyruvate to form acetyl-CoA, releasing CO_2_ and NADH. Subsequently, during aerobic respiration, the TCA cycle coordinates catabolic breakdown of acetyl-CoA, releasing electron flow to the final O_2_ acceptor through a respiratory chain concomitantly generating a transiently stored proton gradient across the inner mitochondrial membrane. This gradient of electric potential energetically fuels oxidative phosphorylation (OXPHOS) mediated by the ATP synthase complex, generating ATP from ADP. Energy from the proton gradient can also be dissipated as heat through passive proton leakage or via uncoupling proteins [4].

**Figure 2:**
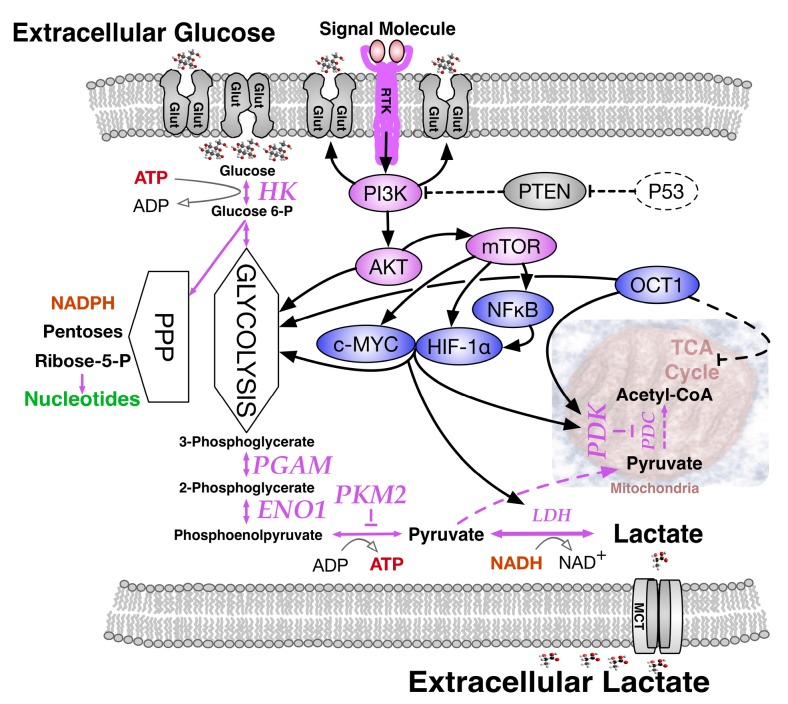
Molecular changes driving the Warburg effect. The shift to aerobic glycolysis in tumor cells reflects multiple oncogenic signaling pathways. Downstream from an active RTK, receptor tyrosine kinase, PI3K, Phosphatidylinositol 3-kinase activates AKT, protein kinase B/Akt strain transforming, stimulating glycolysis by directly regulating glycolytic enzymes. It also activates mechanistic target of rapamycin, mTOR, a protein kinase altering metabolism in various ways, including enhancement of three key transcription factors; the avian Myelocytomatosis viral oncogene proto-oncogene homologue (MYC); Nuclear factor kappa-light-chain-enhancer of activated B cells (NF-κB); and the hypoxia-inducible factor 1 alpha (HIF-1α), to promote a hypoxia-adaptive metabolism. NF-κB subunits bind and activate the *HIF-1α* gene. HIF-1α increases expression of glucose transporters, GLUT, glycolytic enzymes and pyruvate dehydrogenase kinase, PDK, that blocks pyruvate dehydrogenase complex, PDC driven entry of pyruvate into the tricarboxylic acid, TCA cycle. Transcription factor MYC cooperates with HIF-1α to activate several genes encoding glycolytic proteins, yet also stimulates mitochondrial biogenesis whilst inhibiting mitochondrial respiration, favoring substrates for macromolecular synthesis in dividing cells. Tumor suppressor p53 ordinarily opposes the glycolytic phenotype via Phosphatase and Tensin homolog, PTEN but loss of p53 function (dashed line) is frequent in tumor cells. Octamer binding protein 1 (OCT1) activates transcription of genes that drive glycolysis and suppress oxidative phosphorylation. Change to the pyruvate kinase M2, PKM2 isoform affects glycolysis by slowing the pyruvate kinase reaction, diverting substrates into an alternative biosynthetic and reduced nicotinamide adenine dinucleotide phosphate, (NADPH)-generating pentose phosphate pathway, PPP. Phosphoglycerate mutase, PGAM and α-enolase, ENO1 are commonly upregulated in cancer as is the pyruvate kinase M2 isozyme, PKM2 allowing a high rate of nucleic acid synthesis, especially in tumor cells. Lactate dehydrogenase, LDH enhances production of lactate, a signaling molecule that can stabilize HIF-1α and accumulate in the tumor microenvironment via Monocarboxylate Transporters, MCT, nourishing adjacent aerobic tumor cells that convert lactate to pyruvate for further metabolic processing. Solid black arrows, influenced targets; solid purple arrows, metabolite transition pathways; dotted purple arrows, reduced metabolite transition pathways; thin solid black arrow, coenzyme transition; dotted T-bar, inhibition.

Appreciating the essential relationship between oxygen and growth at the cellular level, Otto Warburg and co-workers compared normal liver tissue to corresponding cancer cells and in 1924 described a surprising outcome, namely, the Warburg effect [5,6]. Measuring O_2_ consumption in thin tissue slices metabolizing glucose, they observed that although the respiration of liver carcinoma tissue slices was 20% less than that of normal tissue, about ten-fold more glucose was metabolized than expected. Moreover, the amount of lactic acid, the glycolysis product, was two orders of magnitude higher in cancer cells than in the normal tissue. When the Warburg effect was discovered, an increased glycolysis in cancer cells under aerobic conditions was misinterpreted as evidence for respiration damage. However, we now understand that it reflects an altered regulation of glycolysis in relation to mitochondrial function. The Warburg effect actually comprises a complex collection of contributory changes in gene expression and respiratory function (Figure 2) that include: (i) high glucose transporter expression; (ii) high hexokinase expression; (iii) high pyruvate kinase muscle (PKM2) expression; (iv) expression of phosphoglycerate mutase (PGAM) that allows pyruvate production without ATP generation; (v) high pyruvate dehydrogenase kinase (PDK) levels; and (vi) high expression of specific transcription factors, principally MYC, HIF-1α, NF-κB and OCT1 that sustain the Warburg effect [7]. 

Hexokinase catalyzes the first step in glycolysis by converting glucose to glucose-6-phosphate, making it available for metabolism via the pentose phosphate pathway, or glycolysis and the TCA cycle. Two predominant PKM isoforms are generated from the same gene by different splicing: the fetal form PKM2 uses exon 10, while the adult form PKM1 uses exon 9. The PKM2 protein, often aberrantly expressed in cancer cells, is subject to post-translational phosphorylation of a tyrosine residue that dramatically reduces its ability to convert phosphoenol pyruvate to pyruvate, thereby slowing the TCA cycle via precursor starvation [8]. Though slowed, the TCA cycle remains operational and pyruvate is still produced, but subsequent events conspire to its enhanced conversion to lactate. PGAM activity occurs only in the presence of PKM2 and governs an alternative pathway that converts phosphoenol pyruvate (PEP) to pyruvate without using pyruvate kinase and without producing ATP. In particular, proteomic analysis indicated that PEP, the cellular substrate for pyruvate kinase, contributed to PGAM His-11 phosphorylation to activate its catalytic site [9]. In addition, PDK phosphorylated the pyruvate dehydrogenase complex (PDC) to inactivate PDC, preventing the conversion of pyruvate to acetyl-CoA. Thus, enzyme kinetics for alternative pyruvate use, such as lactic acid production, is improved. Among several transcription factors maintaining the Warburg effect (mainly driven by MYC and HIF-1α with loss of regulatory p53 function) [10] those responding to low oxygen levels are highly significant, since low oxygen conditions (5% O_2_ as opposed 18–20% ambient O_2_) improve blastocyst stage embryo culture in a number of species [11].

Beyond describing how the Warburg effect favors anabolic metabolism, this review will focus on its establishment by the diverse mechanisms hinted at above; chosen adaptive responses that enforce oncogenic events, or stochastically acquired deterioration of mitochondrial function. Collectively, these perspectives provide important clues for understanding the significance of the Warburg effect within complex pathologies and extreme contexts such as space travel. 

## 2. Paradoxes of Efficiency within Perpetual Pyruvate Pathways

A dividing cell would assumedly have high energy requirements, but a paradox of the Warburg effect was that its mechanism for providing free energy in the form of ATP was less efficient. However, this presumably provided a selective advantage to actively dividing cells, given an association between aerobic glycolysis and proliferation across species. Beyond homeostatic energetic requirements, growth and cell division require anabolic processes. The Warburg effect may be an evolutionary conserved mechanism for balancing ATP production with biomass production.

Focus on metabolic regulation in cancer provided a unifying theory for understanding interactions between prominent oncoproteins and tumor suppressors that deregulate glycolysis. The metabolic perspective extended tumorigenesis beyond a cumulative cascade of growth signal activation and tumor suppressor gene inactivation, introducing conceptually useful driving forces exploitable for cancer therapy.

Typically, the conventional paths for glucose metabolism and TCA cycle generate in adult tissues a large amount of ATP (up to 36 molecules of ATP per molecule of glucose). Pyruvate can also be reductively metabolized to organic acids or alcohols (e.g., lactic acid or ethanol) by anoxic glucose fermentation, a far less efficient pathway for generating ATP (only two molecules of ATP per molecule of glucose). Despite the much lower relative yield, ATP production rate remains high if the glucose supply is abundant. This alone does not explain the advantage to aerobic glycolysis, because most ATP is derived from other sources and is not limiting in proliferating cells. Consumption, rather than production of ATP is needed to overcome a high ATP/AMP ratio that would ordinarily inhibit key rate-limiting steps in glycolysis. Moreover, there is an associated greater need for NADPH to regulate glycolytic transcription factors and support fatty acid synthesis. Mechanisms that increase ATP consumption drive glycolytic conversion of glucose to lactate. This may again seem paradoxical if one imagines that generation of glycolytic intermediates for biosynthesis of macromolecules would be more helpful than excretion of carbon as lactate. Yet this may serve as a regulatory buffer for the process of biomass production, allowing cells to increase biosynthesis during cell proliferation only when precursor concentrations are appropriately abundant.

Pyruvate, the final product of glycolysis, has three main fates in mammalian cells: (1) conversion to lactic acid via lactate dehydrogenase (LDH); (2) conversion to alanine via alanine aminotransferase with concomitant conversion of glutamate to α-ketoglutarate; (3) conversion to acetyl-CoA in mitochondria via the pyruvate dehydrogenase (PDH) complex to enter the TCA cycle. The Warburg effect influences pyruvate fate by increasing its provision for anabolic processes. A high glycolytic flux in proliferating cells saturates the maximum PDH activity, leaving excess pyruvate for the action of LDH and alanine aminotransferase. Some cancer cells secrete conspicuously large amounts of alanine, that frequently parallel cellular lactate levels and may reflect a consequence of using glutamine to generate NADPH [12]. Conceptually, maximizing pathway flux maximizes ATP yield when TCA cycle enzyme production is limiting. Relatively high costs of enzyme synthesis ultimately decrease the ATP production rate. Accordingly, given choice between higher-flux, low-yield metabolic pathways (aerobic glycolysis) and lower-flux, high-yield pathways (invoking the TCA cycle), maximizing pathway flux surpasses reallocating proteins away from glycolytic processes towards the TCA cycle and respiratory chain enzymes [13]. Improved ATP economy, rather than *de novo* generation of biomaterial, may explain the maintenance of low-yield pathways such as the Warburg effect in tumor cells. Finding the Warburg effect also in non-replicating striated muscle tissues is consistent with this interpretation.

## 3. The Need for NADPH and Diversified Carbon Sources

Cells in metabolically stressed microenvironments may find themselves short of glucose as well as oxygen. Serum starved human fibroblasts can switch to anaerobic metabolic pathways that mimic the Warburg effect [14]. The AMP-activated protein kinase (AMPK), a known cell metabolism sensor, is activated by phosphorylation during situations of metabolic stress. AMPK regulates levels of NADPH, a key coenzyme that removes dangerous reactive oxygen species (ROS) in anabolic reactions, crucial to several metabolic processes for cell survival [15]. Ordinarily, the glucose-utilizing oxidative phase of the pentose phosphate pathway (PPP) generates most of the NADPH. When glucose is limiting, AMPK can inhibit activity of the NADPH-consuming metabolic enzyme acetyl-CoA carboxylase to indirectly maintain intracellular NADPH levels. NADPH is mostly a reducing agent in anabolic reactions, whilst reduced nicotinamide adenine dinucleotide (NADH), which differs by a phosphate group that allows distinction by a different set of enzymes, is principally involved in catabolic reactions. When oxygen is absent or in short supply, LDH catalyzes the conversion of pyruvate to lactate with concomitant interconversion of NADH to its oxidized form, (NAD^+^). Regeneration of NAD^+^ is necessary for continued flux through glycolysis and mediates conversion of glyceraldehyde-3-phosphate to 1,3-bisphosphoglycerate by glyceraldehyde 3-phosphate dehydrogenase (GAPDH). This is the sixth step of glycolysis that uses NAD^+^ to produce NADH for maintaining the cellular redox state required for regulating gene expression. Both reduced forms of NAD^+^ (NADH and NADPH), activate transcription factors such as the transcriptional regulator C-binding protein involved in cell growth, differentiation and transformation [16]. In a counterpoised manner, the oxidized forms (NAD^+^ and NADP^+^) can inhibit transcription factor binding to DNA [17]. Indicative of anabolic requirements for lipid biosynthesis, as many as 14 molecules of NADPH are required for generating each molecule of palmitoyl-CoA and 26 molecules of NADPH are required for cholesterol. Cytosolic isocitrate dehydrogenase (IDH1) generates NADPH by converting isocitrate to α-ketoglutarate. IDH1 may be a vulnerable requirement for particular cancer cells [18] although other cancers bear dominant inhibitor mutations of IDH1 and presumably have other means for NADPH production. In sum, lactate production and its by-product NAD^+^ can enhance both glycolytic flux and incorporation of glucose metabolites into biomass, to allow faster cell growth. In situations such as embryogenesis, immune response and wound healing, when nutrients are abundant, rapid cell division provides a more significant selective advantage than efficient carbon utilization.

**Figure 3:**
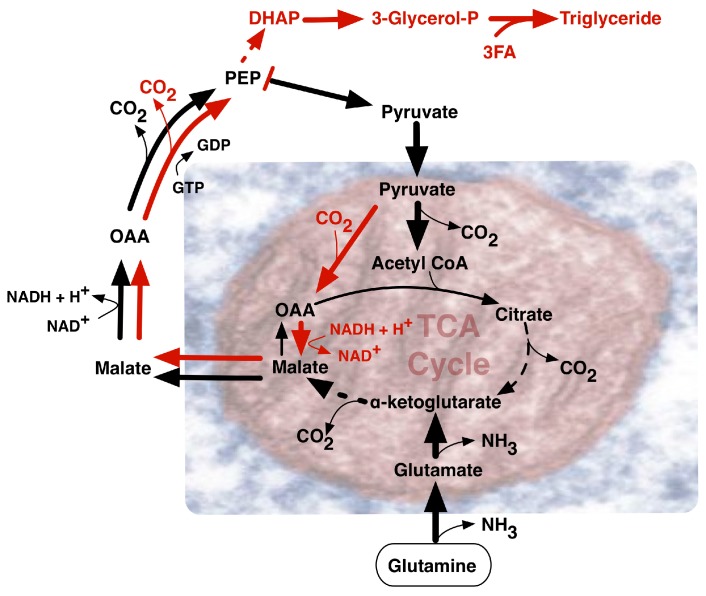
Glutamine metabolism: The influence of anaplerosis (entry of glutamine into the TCA cycle), cataplerosis (removal of glutamine as malate) and glyceroneogenesis (marked red). Mechanisms by which glutamine is metabolized for energy involve entry of glutamine into the TCA cycle (anaplerosis), balanced by its removal (cataplerosis) as malate. Malate can be subsequently converted to oxaloacetate (OAA) and then to phosphoenolpyruvate (PEP) via the cytosolic enzyme phosphenolpyruvate carboxylase (PEPCK). PEP can be converted to pyruvate by pyruvate kinase for entry into the TCA cycle as acetyl-CoA or transamination to alanine. The pathway of glyceroneogenesis in which carbon from sources other than glucose or glycerol contributes to the formation of l-glycerol-3-phosphate (3-Glycerol-P) for conversion to triglycerides, involves a balance of anaplerosis (entry of OAA synthesized from pyruvate via pyruvate carboxylase) and cataplerosis (removal of intermediates to support the synthesis of glyceride-glycerol). Reduction of glycolysis-derived dihydroxyacetone phosphate (DHAP) to 3-Glycerol-P provides cells with the activated glycerol backbone needed to synthesize new triglycerides with fatty acids (FA). Solid arrows, anaplerosis pathways; red arrows, glyceroneogenesis pathway; dotted arrow, mitochondrial cataplerosis pathway.

One additional route supplying high demand for NADPH in proliferating cells, involves NADPH synthesis from glutamine metabolism (Figure 3) via its oxidation to malate with subsequent activity of malate dehydrogenase generating pyruvate and NADPH [12]. Notably, glutamine uptake and metabolism proved to be regulated by the *MYC* oncogene [19]. *MYC*-transformed cells may become dependent on glutamine and exhibit elevated expression of glutamine transporters and glutamine catabolic enzymes. Alternatively, in less glutamine dependent MYC-deficient tumors, IDH1 activity may be the main source of NADPH, although further undefined sources remain likely. Glutamine is the most abundant amino acid in human plasma and a major contributor to the replenishment of TCA cycle intermediates (anaplerosis). Such replenishment sustains TCA cycle intermediate efflux (cataplerosis) maintaining precursors for many non-essential amino acids for biomass synthesis. Fatty acids, such as acetyl-CoA, along with nitrogen molecules in nucleotides, amino acids and amino sugars, can also be derived from glutamine, as well as glucose [20], through a process coined glutaminolysis that contributes to glyceroneogenesis (Figure 1). Increased levels of acetyl-CoA induced by MYC may not only enter the TCA cycle, but also serve as a donor for histone acetylation associated with gene activation [21]. MYC can activate the expression of glutaminase 1 (GLS1) that deamidates glutamine to produce glutamate [19]. Conversion of glutamine to glutamate fuels folate metabolism since addition of glutamates to folates increases the retention of folates within cells, promoting GLS1 activity. Under low oxygen conditions, reductive glutamine metabolism can contribute significantly to lipid biosynthesis. As a reciprocal carbon source alternative to glucose in diploid fibroblasts, glutamine could provid 30% of the ATP energy requirement when cells were cultured in standard 5.5 mM glucose-containing medium with increased glutamine utilization in lower concentrations of glucose [22]. In rapidly proliferating HeLa cells cultured in 10 mM glucose-containing medium, glutamine still provided over 50% of the ATP requirement, emphasizing its key role in supporting a proliferative metabolism.

## 4. The Warburg Effect in Normal and Cancer Cells; Deriving the Choice, Chain or Chance Perspective

Though conspicuous in cancer, a fundamental question of the original Warburg effect was whether it reflected an aberrant condition or an adaptive feature to be also found in normal cells. Viewed as a “choice”, accumulating evidence indicated that the Warburg effect was probably a metabolic pathway advantageously chosen by normal cells during early development. Alternatively, many tumorigenic events are strongly linked to regulation of metabolism, and certain critical mutations may “chain” cancer cells to the Warburg effect pathway. “Chance” events accompanying ageing can include mutations that impair mitochondrial function [23] often encouraging a metabolic drift towards aerobic glycolysis.

**Figure 4:**
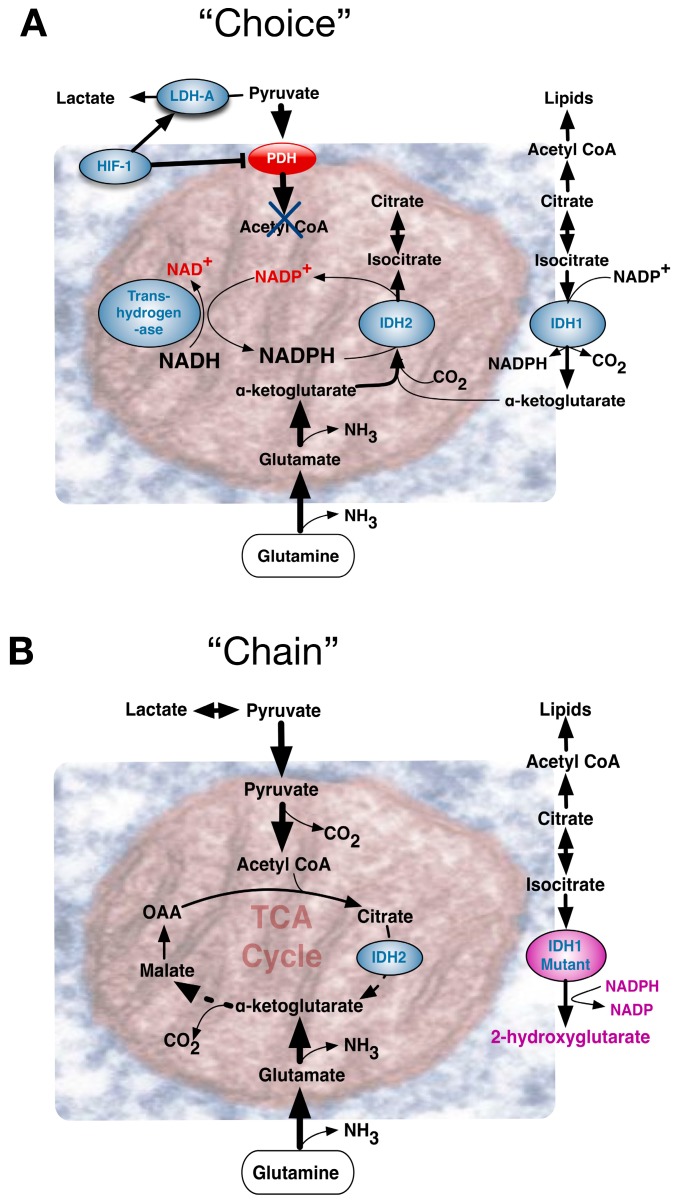

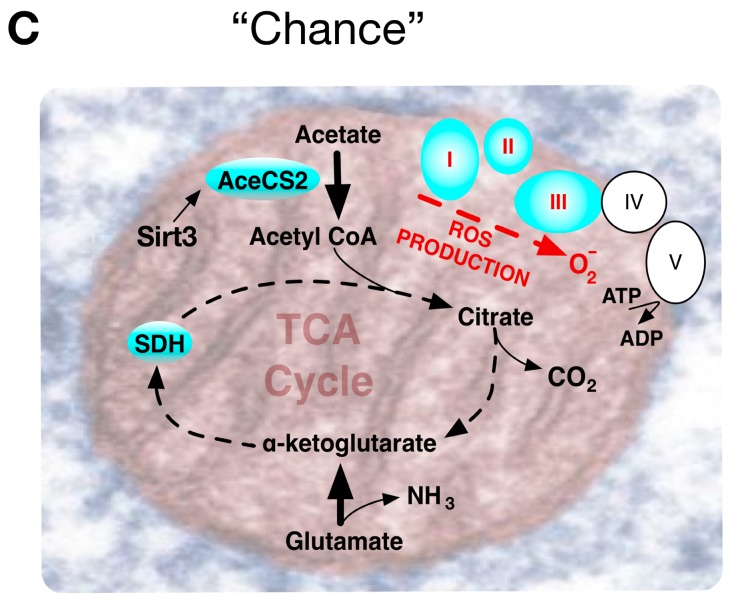
Mechanisms influencing the mitochondrial metabolism and the Warburg effect. (**A**) Environmentally driven metabolic “choice”. In cells proliferating under hypoxic pressure with activated HIF-1, the electron transport chain is inhibited because of the lack of oxygen as electron acceptor. HIF-1-induced inactivation of PDH-1 helps ensure that glucose is diverted away from mitochondrial acetyl-CoA-mediated citrate production. The alternative pathway for maintaining citrate synthesis involves reductive carboxylation, thought to rely on a reverse flux of glutamine-derived α-ketogluturate via isocytrate dehydrogenase-2 (IDH2). The reverse flux in mitochondria can be maintained by NADH conversion to NADPH by the mitochondrial transhydrogenase, with the resulting NADPH driving α-ketoglutarate carboxylation. Citrate/isocitrate exported to the cytosol may be metabolized oxidatively by isocytrate dehydrogenase-1 (IDH1), and contributes to the production of cytosolic NADPH. (**B**) Mutational events that “chain” cells to particular metabolic pathways. Oncogenic IDH1 and IDH2 mutations can cause gain of function in cancer cells. Somatic mutation at a crucial arginine residue in cytoplasmic IDH1 or mitochondrial IDH2 are frequent early mutations in glioma and acute myeloid leukaemia which cause an unusual gain of novel enzymatic activity. Instead of isocitrate being converted to α-ketoglutarate, with production of NADPH, α-ketoglutarate is metabolised to 2-hydroxyglutarate with the consumption of NADPH. 2-hydroxyglutarate can compete for α-ketoglutarate-dependent enzymes including histone demethylases and DNA hydroxylases, thereby altering both metabolism and epigenetic phenotypes. (**C**) “Chance” deterioration events in ageing. SIRT3 regulates multiple pathways involved in energy and ROS production. Some mitochondrial metabolic processes activated by SIRT3 include acetate metabolism-mediated direct deacetylation of acetyl-CoA synthetase (AceCS2), the TCA cycle via direct deacetylation of SDH, and ROS production induced by direct acetylation of the respiratory chain complexes I, II and III. Thin solid black arrow, alternative reductive carboxylation pathway; Solid black arrow, TCA cycle pathway; dotted arrow, alternative TCA cycle pathways; red dotted arrow ROS production pathway.

Accordingly, influential events can be conceptually considered to reflect A, Environmentally driven metabolic “choice”, or B, driving mutational events that “chain” cells to particular metabolic pathways, or C, “chance” deterioration events of ageing (Figure 4). Comparing the Warburg effect in different circumstances, requires appreciation of its influence in contexts that diversely impinge upon an apparently similar phenotype. Metabolic pathways need to be immediately and dynamically responsive to an organism’s situation, with the combination of the rate of flux and yield efficiency determining the ultimate outcome. 

### 4.1. The Warburg Effect by Choice; an Adaptive Response to Oxygen and Nutrient Restrictions

Supporting the idea that early fetal and placental development is primarily an anaerobic process, the anatomical features of the first trimester gestational sac limit rather than facilitate oxygen transfer to the fetus. Additionally, increased levels of antioxidant molecules are found in the exo-coelomic cavity when oxygen free radicals have the greatest potential for harmful teratogenic effects [24]. An in vivo Xenopus model of embryonic retinal development unequivocally showed that dividing progenitor cells relied more on glycogen to fuel aerobic glycolysis than non-dividing differentiated cells, demonstrating that the Warburg effect was a feature of physiological cell proliferation [25]. Early ex vivo studies in human embryonic stem cells paradoxically showed either increased [26] or decreased oxidative phosphorylation [27] in response to experimentally defined nutrient or oxygen conditions. However, the prevailing view of comprehensive metabolomic studies was that the stem cell redox status changed during differentiation, with the Warburg effect influencing pre-implantation embryo development [28]. Dramatic metabolic differences accompany distinct pluripotent states in early embryogenesis. Embryonic stem cells (ESC) of the inner cell mass of pre-implantation embryos show bivalent energy production, maintaining a relatively high ATP content. In contrast, post-implantation epiblast stem cells (EpiSC) have lower mitochondrial respiratory capacity, despite a more developed mitochondrial content, due to low expression of cytochrome c oxidase. Early mammalian embryo metabolism presents unique features, significantly different from proliferating somatic cells. Pre-implanted cleavage-stage embryos need to mainly replicate DNA and plasma membranes rather than entire cells and during cleavage from a single cell to a blastocyst, cells get progressively smaller. At the blastocyst stage, the somatic cell size is reached, and cells need to replicate fully prior to the next rapid cell division. This requires a unique metabolism that is more closely equivalent to the Warburg effect in cancer. Just as hypoxia has been shown to influence tumorigenesis, HIF-1α sufficed to drive the metabolic switch from an ESC to an EpiSC-like stage [29].

Cancer cells evolve by acquiring a selective advantage for survival and proliferation in a challenging tumor microenvironment. As a tumor outgrows the diffusion limit of local blood supplies, its cells encounter hypoxia. Under such circumstances, a Warburg effect in cancer cells may be considered a chosen reversible adaptation in response to local hypoxia. The stabilized hypoxia inducible transcription factor complex, a heterodimer of oxygen-dependent HIF-1α and constitutively expressed HIF-1β, activates a transcriptional program accommodating cells to hypoxic stress through diverse compensatory mechanisms [30]. Befitting choice for a Warburg effect, both decreased dependence on aerobic respiration and metabolism shifts towards glycolysis become advantageous. These changes are mediated by increased expression of glucose transporters, glycolytic enzymes and inhibitors of mitochondrial metabolism (Figure 4A). At the same time, HIF-induced molecules such as vascular endothelial growth factor (VEGF) stimulate angiogenesis to restore blood flow and oxygen supply. Nonetheless, early blood vessel growth within a growing tumor’s poorly organized tissue architecture is often sub-optimal and may not deliver blood effectively, alleviating the oxygen requirements only partially. Adaptive responses of tumor cells to hypoxic conditions in the tumor niche have a persistent influence on tumor growth. HIF-1α has two other isoforms HIF-2α and HIF-3α, as does the HIF-1β subunit, aka the aryl hydrocarbon receptor nuclear translocator (ARNT1, Arnt2 and Arnt3). Under oxygen concentrations below 6% O_2_, the HIF α-subunit of short 5-minute half-life, was stabilized upon activation and transport to the nucleus where it dimerized with the β subunit to bind hypoxia responsive elements in target genes [31]. HIF-1α and HIF-2α, with distinct structure within the N-terminal transactivation domain, play key roles in hypoxia-induced cellular responses. Whereas HIF-1α predominantly induces glycolytic pathways, HIF-2α regulates genes important for cell cycle progression and maintenance of stem cell pluripotency, including MYC and the stem cell factor OCT-3/4. Although HIF-1α and HIF-2α both exert similar induction of the angiogenic *VEGF* gene, they may exert opposite effects on other angiogenic factors, e.g., HIF-1α decreases IL-8 expression whereas HIF-2α increases IL-8 mRNA and protein levels [32].

Fluctuating oxygen levels would favor selection of tumors that constitutively upregulate glycolysis, yet the observed heterogeneity of HIF expression within tumor cells would suggest that this signaling pathway is still coupled to oxygen levels in most tumors. MYC and HIF family members both recognize related DNA-binding sequences in the promoters of glycolytic target genes, suggesting a coordinated regulation of expression. But when controlling mitochondrial function, the interactions between MYC and HIF proteins are often antagonistic. HIF-1α can activate expression of pyruvate dehydrogenase kinase 1 (PDK1), which subsequently phosphorylates and inhibits pyruvate dehydrogenase, preventing glucose-derived acetyl-CoA from entering mitochondria and the TCA cycle. Such pathways may restrict the positive role of MYC and thereby restrain cell growth and oxygen consumption in hypoxic conditions. Thus, a complex set of biochemical interactions between MYC and HIF dictates outcomes depending on the promoter context of target genes, the transformed state of the cells and oxygen tension [33].

Emphasizing HIF importance for regulating cell outcomes such as enhanced proliferation, patients with Von Hippel Lindau disease (pVHL) who lack the protein mediating HIF1α degradation, are predisposed to develop a range of highly vascularized tumors [34]. In addition, patients with Chuvash polycythemia, a hereditary disorder of increased sensitization to hypoxia, show hepatic hyper-proliferation and a correspondingly increased organ volume [35]. However, up-regulation of the HIF signaling pathway needn’t entirely account for the Warburg effect per se, since many additional events of transformation can directly enforce a Warburg effect metabolism. Nutrient deprived cancer cells can resort to glycolysis for ATP production, evoking pathways involving ROS production and AMPK phosphorylation leading to PDK activation and enhancement of the Warburg effect [36].

### 4.2. The Warburg Effect Chained to Oncogenic Events

A strong association between cancers and the Warburg effect allows the metabolic label ^18^F-deoxyglucose to localize tumors via positron emission tomography (FDG-PET). More than simply an indirect metabolic response, the Warburg effect may represent an integral metabolic necessity for cell proliferation towards which multiple oncogenic events conspire [37]. Many well-established irreversible molecular oncogenic changes can be linked to the establishment of a Warburg effect. Therefore, tumor cells may become effectively “chained” to aerobic glycolysis for enhancing proliferation-favorable NADPH production and acetyl-CoA flux to the cytosol for lipid biosynthesis. For example, over-expression of the receptor tyrosine kinase (RTK), human epidermal growth factor 2 (HER-2) by gene amplification, is linked to an aggressive cancer cell subtype by inducing up-regulation of a key lipogenic enzyme, the fatty acid synthase (FASN). This allows rapid response to changes in the flux of lipogenic substrates (e.g., NADPH and acetyl-CoA) and lipogenesis products (e.g., palmitate). In addition, HER-2 can also up-regulate PPARγ. This promotes adipogenesis and lipid storage to avoid endogenous palmitate toxicity by securing palmitate in fat stores rather than allow its negative feedback on FASN function [38]. The K-ras oncogene can modulate the cyclic adenosine monophosphate-dependent protein kinase (cAMP/PKA) signaling pathway, ultimately influencing mitochondria through decreased mitochondrial complex I activity and reduced ATP formation. This makes, *K-ras* mutant cells more sensitive than normal cells to glucose withdrawal [39]. Estrogen receptors directly bind to the promoters of many genes encoding glycolytic enzymes and these can interact synergistically with the proto-oncogene transcription factor MYC to drive the Warburg effect in cancer cells [40].

Few tumorigenic pathways influencing glucose metabolism are more important than the phosphoinositide-3-kinase (PI3K) signaling pathway. Downstream activation of protein kinase AKT induces glucose transporter *GLUT1* gene expression and prevents internalization of GLUT1 protein to maintain cell surface levels for higher glucose uptake. AKT activation also promotes flux through glycolysis, and numerous cancer cell mutations that constitutively activate PI3K, bypassing need for growth signals, ultimately promote aerobic glycolysis [12]. The first reported direct link between oncogene activation and altered glucose metabolism involved MYC induced LDH-A expression [41]. In addition to altering glutamine metabolism, *MYC* oncogene driven cell cycle progression, could induce genomic instability and also promote transcription glycolytic enzymes as well as glucose transporters GLUT-1, GLUT-2 and GLUT-4 [42]. MYC-stimulated LDH-A production could become crucial for proliferation of MYC-dependent tumors [43]. MYC could also influence membrane biosynthesis via enhanced synthesis of fatty acids from glutaminolysis. 

An intricate associate between transcription factors and glycolysis, modulated by the carbon source, is coordinated by glucose sensing mediated by MondoA, a basic helix-loop-helix leucine zipper (bHLHZip) transcriptional activator functionally similar to MYC [44]. Resembling MondoA, a further family member Carbohydrate Response Element Binding Protein (ChREBP/MondoB/WBSCR14) can dimerize with yet another bHLHZip protein called Max-like protein x (Mlx). Regulation of their activity is largely controlled by intracellular location with accumulation in the nucleus subject to different glucose-derived metabolites. MondoA:Mlx heterodimers accumulate in the nucleus in response to glucose 6-phosphate (G6P), whereas ChREBP:Mlx accumulates in response to the pentose phosphate intermediate xylulose 5-phosphate [45]. Thus, an adaptive transcriptional response to glucose and ATP levels is coupled to activation of metabolic genes, including glycolytic enzymes. MondoA and ChREBP also regulate expression of thioredoxin interacting protein (TXNIP) that has a broad range of activities, including glucose homeostasis [46] and inhibition of thioredoxin redox activity, elevating ROS levels that influence key target genes that help control cell growth. Interdependency is such that, MondoA was responsible for most of the glucose-induced transcription in an epithelial cancer cell line [47].

Providing an intriguing link with the highly significant tumor suppressor gene *p53*, MondoA knockdown stimulated cell growth in *p53*-deficient cells, whereas ChREBP knockdown reduced cell growth in *p53* wild-type cells, suggesting that the functional output of MondoA and ChREBP transcriptional responses was subject to p53 status [48]. Indeed, the pleiotropic p53 has been shown to influence and respond to metabolic changes through several cancer cell-antagonizing mechanisms, including promotion of apoptosis, senescence and DNA repair. Moreover, p53 could promote oxidative phosphorylation and dampen glycolysis hence interfering with cell growth and autophagy. As such, *p53* mutations unite oncogenic transformation and altered metabolism [49].

Despite all the above, tumors often retain a degree of metabolic flexibility. In a mass spectrometry-based global metabolomic study, Abu Dawud et al., directly compared human embryonic stem cells (hESCs) and embryonal carcinoma cells (hECCs). As might be expected for a metabolism of choice, undifferentiated hESC utilized aerobic glycolysis rather than OXPHOS, with both normal and cancer cell types expressing equivalent amounts of glycolysis intermediates [50]. Yet the study also highlighted metabolic differences between the two cellular types. Proliferation in hECCs involved higher levels of metabolites. hESCs could be induced to differentiate by depletion of OCT4, that induced gene expression for key mitochondrial respiratory chain proteins, including NDUFC1, UQCRB and COX. Many OXPHOS components were already expressed at the mRNA level in undifferentiated hESC, as though these cells were “poised” to enter the TCA cycle. Moreover, TCA cycle intermediates were enriched in hECC relative to hESC, implying that cancerous hECC may not rely solely on the Warburg effect yet have some dependency on both pathways. Curiously, gene-knockout mitochondrial DNA depleted B16 mouse melanoma cells, restricted to an exclusively glycolytic metabolism, could form primary subcutaneous tumors, yet no longer formed lung tumors when injected intravenously in NOD/SCID recipient mice. This suggested that ROS byproducts of mitochondrial aerobic metabolism may be required for tumor metastasis [51]. This needn’t contradict the important contribution of a glycolytic pathway for metastasis [52] but highlights that beyond toxic mutagenic effects, ROS signaling molecules influence adaptive responses to harsh microenvironments, including regulating of cell adhesion via integrins [53]. Certainly, ideas that cancer cells are strictly driven to one particular mode of metabolism is likely to be an over-simplification, and a more complex alternating metabolic scenario seems likely [54]. Surrounding cells that comprise the tumor microenvironment should be also be considered important contributors [55,56]. 

Though most cancers retain a normal OXPHOS capacity [57], the Warburg Effect can be imposed by links to TCA cycle protein mutations. Fumarate hydratase (FH) catalyzes the hydration of fumarate into malate and FH germline mutations are implicated in hereditary leiomyomatosis and renal cell cancer (HLRCC). FH loss leads to fumarate accumulation that in turn activates hypoxia-inducible factors at normal oxygen tensions. The metabolic consequences of a crippled TCA cycle include a potential NADH shortage, yet *Fh1* deficient cells retain significant mitochondrial bioenergetic activity through compensatory mechanisms involving the biosynthesis and degradation of haem [58]. Other mitochondrial proteins can be mutated in a very specific manner. Frequently, in low-grade gliomas, single amino acid substitution mutations for an arginine residue in the active site of IDH1 and IDH2 evoked production of a new metabolite, 2-hydroxyglutarate (2-HG). Exactly how this exerts oncogenic effects remains to be resolved [59], but it supports the concept of “onco-metabolite” (Figure 4B).

### 4.3. The Warburg Effect Evolved by Chance during Ageing

Most experimental mitochondrial OXPHOS measurements have employed isolated mitochondrial complexes that needn’t entirely correspond with the functioning of mitochondria in vivo. For example, the respiratory chain complex IV enzyme cytochrome oxidase (COX4), with twice the free energy yield of earlier complex I and III respiratory chain steps, represented a rate-limiting step of respiration in intact cells [60], but not in isolated mitochondria [61]. Again, unlike complexes I and III, COX4 converts O_2_ into water without forming ROS. Abnormally high levels of ROS can directly induce genomic instability and increase HIF-1α levels, promoting metabolic programming towards the Warburg effect. Notably, Sirtuin gene *Sirt3*, a mammalian NAD-dependent protein deacetylase, homolog of *Saccharomyces cerevisiae Sir2* that regulates yeast life span, deactivates mitochondrial target proteins in critical pathways associated with age-related diseases. Sirtuin deacetylase activity on lysine residues of numerous mitochondrial substrates induces fat oxidation, amino-acid metabolism and electron transport. Transgenic mice lacking *Sirt3* have increased ROS levels, caused by both decreased electron transport and decreased detoxification activity of MnSOD. *Sirt3* reveals a connection between mitochondrial metabolism promoting the Warburg effect, tumorigenesis and induction of oxidative stress associated with randomly accumulated lesions causing degenerative diseases and ageing [62] (Figure 4C).

Notably, several mitochondrial ribosomal and transfer RNAs plus 13 proteins representing subunits of complex I, III, IV and V proton pumps are encoded by mitochondria-specific DNA (mtDNA). mtDNA is transcribed and translated independently from nuclear DNA, with a relatively fragile exclusively maternal inheritance, suffering a ten-fold higher mutation rate than nuclear DNA. The enhanced genetic fragility may explain maternally inherited mitochondrial diseases and ageing may be partly due to somatic mutations of mtDNA leading to impaired COX4 activity, impaired synthesis of ATP and reduced cell energetics with age. Maintaining adequate flux and COX4 activity reduces ROS mediated double strand breaks in mtDNA, with subsequent mtDNA repair vulnerable to introduction of mitochondrial DNA deletions [63]. Accordingly, mitochondrial DNA deletions have been associated with biochemical defects in neurodegenerative diseases like Alzheimer’s disease [64]. However, direct evidence for this mechanism as an explanation for ageing and broader spectrum metabolic diseases has been elusive. Recent investigations in cultured human senescent myoblasts [65] and transgenic mouse studies using mtDNA exchange technology [66] lacked evidence that patients with mitochondrial diseases due to pathogenic mtDNA mutations develop premature ageing. Nonetheless, the trans-mitochondrial mice did develop diabetes and lymphomas, so significant mitochondrial respiration defects may lead to over-production of ROS with pathological consequences including ROS impairing glucose incorporation into insulin-targeted organs [67] or insulin secretion from pancreatic β cells [68]. 

## 5. The Warburg Effect and Diabetogenesis

A simulated Mars mission over 105 days, as an example of extreme conditions pressuring the human organism, indicated that environmental stress would exert considerable metabolic stress [69], and space flight perturbations in insulin sensitivity led to subclinical type 2 diabetes-like symptoms [70]. However, to what extent chronic pathological effects would be exacerbated during prolonged space flight missions is not known. Nonetheless, reference to what is understood about molecular events and pathways underlying type 2 diabetes is likely to be helpful for assessing the most pertinent measurements for monitoring health in space and preventative measures. 

A challenging concern for understanding complex metabolic diseases such as type 2 diabetes mellitus (T2DM) is discrimination between cause and effect, given the close reciprocal relationship between metabolic pathways and disease phenotypes. Nonetheless, foundational metabolic aspects from lessons learned about the Warburg effect in development, cancer and ageing can be applied with a choice, chain or chance perspective to explore relevance in T2DM. Here, we apply this perspective to highlight pivotal events that may serve as useful diagnostic markers or therapeutic targets.

The enigmatic T2DM phenotype reflects multifactorial mechanisms responsible for the phenotypes of impaired insulin secretion from pancreatic β cells and insulin resistance in the major target tissues, such as skeletal muscle. An alarmingly high demographic incidence of T2DM, associated with sedentary lifestyles [71] and inappropriate carbohydrate diets [72] urges need to identify and characterize the repertoire of key regulatory molecules.

### 5.1. Lessons for T2DM from a Warburg Effect through Normal Cell Metabolic Choice

When the Warburg effect resulted from a contextual choice, gene expression changes following hyperglycemic treatment of early stage bovine blastocysts showed metabolic changes common to diabetes and cancer. This may help explain why diabetic hyperglycemia is often associated with cancer predisposition [73]. In diabetes, hyperglycemia increases intracellular glucose and its conversion to sorbitol by aldose reductase decreases NADPH levels. Hyperglycemic induction of this polyol pathway proves harmful because the lack of NADPH limits glutathione reduction that ordinarily protects cells from oxidative stress [74]. Blastocysts treated with excessive glucose (5 mM) showed increased expression of glutathione peroxidase 8 (GPX8), an antioxidant enzyme that reduces H_2_O_2_ into water by oxidation of glutathione, implying NADPH depletion may activate a compensatory oxidative stress response. This is similar to the elevation of GPX expression correlated with increased aldose reductase activity found in the pathology of type 1 diabetes [75]. An additional diabetic hallmark of hyperglycemia is activation of the hexosamine pathway. This diverts fructose-6-P from the glycolytic pathway with associated glucosamine-6-P accumulation inducing protein glycosylation of transcription factor Sp1. This induces *SERPINE 1* and *THBS1 gene* expression, both genes being implicated in diabetic pathogenesis. Similar upregulation of *SERPINE1* and *THBS1* in hyperglycemically treated blastocysts likely reflected increased activity of the hexosamine pathway. Underlying diabetic complications is a hyperglycemia-induced uncoupling of OXPHOS, which leads to mitochondrial ROS production inhibiting GAPDH activity, with glycolytic metabolites diverted to stimulate the polyol, AGE, PKC and hexosamine pathways [76]. In agreement, porcine embryos grown in elevated glucose medium showed an early rise in ROS generation and decreased GAPDH activity. Moreover, they also shared the diabetic trait of decreased citrate synthetase activity, responsible for entry of pyruvate-derived acetyl-CoA towards TCA. Decreased TCA activity in high-glucose treated blastocysts can also reflect up-regulation of nuclear receptor PPARγ that regulates lipid metabolism, with subsequent accumulation of oxidized lipids. These are likely to stimulate higher expression of OLR1 (oxidized low-density lipoprotein receptor-1) a scavenger of oxidized lipids, often up-regulated in diabetes, with an important role in pregnancy disorders [77] and tumorigenesis [78].

Matching the growing energy demands of development, blastocysts increase mitochondrial OXPHOS and activate glycolysis and glucose uptake to enhance ATP synthesis. Hyperglycemic conditions increased blastocyst expression of *PDGFC* and *HIF-1α* to promote expression of lactate dehydrogenase A (LDHA) that enhances anaerobic conversion of pyruvate into lactate, with production of NAD^+^. Under these conditions, the blastocysts also increased expression of transketolase 1, an enzyme catalyzing the non-oxidative part of the pentose phosphate pathway in order to enhance glucose consumption and lactate production [79]. Thus, under diabetic conditions, the increased anaerobic glycolysis in embryos could limit ROS generation as a beneficial compensation for the impaired energy metabolism. Despite such similarities with the Warburg effect phenotype of cancer cells, in most cases, early development ultimately avoids a tumorigenic fate. In non-tumorigenic cells, the activity of tumor suppressors such as p53 readily activates expression of proteins mediating an apoptotic mitochondrial death pathway in response to a depressed mitochondrial transmembrane potential. Excessive early embryonic lactate production is associated with aborted gestation [80] emphasizing the detrimental impact of early hyperglycemic stress on pre-attachment embryo survival and blastocyst development.

### 5.2. Lessons for T2DM from an Oncogenically Chained Warburg Effect

A caveat when extending the biochemical events governing the Warburg effect in cancer to other contexts such as diabetes is that cancer cells likely have additional events, such as tumor suppressor mutations, resulting in very different context-dependent metabolic feedback. Thus, key molecular events governing the Warburg effect in tumor cells might not have the same influence in skeletal muscle cells or pancreatic β-cells and vice-versa. Nevertheless, tumor cells can provide an abnormally permissive environment making key control events more significantly apparent. An example is provided by human LDH that catalyzes the reduction of pyruvate into lactate, a watershed event for the switch from aerobic to anaerobic glycolysis. The tetrameric LDH is made up of two subunits, LDHA (aka LDH-M, muscle) and LDHB (aka LDH-H, heart), with isoforms made up of various combinations of these two subunits, e.g., LDH3=LDHA_2_B_2_ whilst LDH5=LDHA_4_, the form most prevalent in the liver and skeletal muscle. In cancer patients, high plasma hLDH5 levels can serve as a malignant cell biomarker, since it is associated with induction by *HIF-1α* and *MYC*, rather than nonspecific cellular damage. Oncogenic tyrosine kinase fibroblast growth factor receptor (FGFR1) directly phosphorylates LDHA subunits, enhancing the formation of active tetrameric LDH5 that binds to NADH substrate. Thus, oncogenic tyrosine kinases activation, a common event in many cancers, promotes the Warburg effect via LDHA, for enhanced tumor growth [81]. Inhibitors preferentially targeting the LDHA isoform in cellular assays, reduced lactate production in HeLa cells and were particularly effective in blocking cell proliferation in hypoxic conditions [82]. In contrast, pancreatic islet β-cells normally express low levels of LDHA and have high glycerol phosphate dehydrogenase activity. Acute experimental over-expression of LDHA in the murine model MIN6 β-cells perturbed mitochondrial metabolism, interfered with normal glucose-derived pyruvate metabolism in mitochondria and inhibited glucose-stimulated insulin secretion [83]. Thus, an LDHA abnormality highlighted by oncogenically “chained” events remained influential in more normal contexts, being more open to a “choice” of feedback.

Another example from cancer metabolism, is related to pyruvate dehydrogenase kinase 1 (PDHK1) which is also inducible by MYC and HIF-1α. It is again activated by tyrosine phosphorylation that induced the binding of ATP and PDC, promoting the Warburg effect with tumor growth [84]. Complementing tyrosine phosphorylation in glucose homeostasis, protein tyrosine phosphatases (PTPs) are key negative feedback regulators of insulin receptor (IR) signaling that effectively inhibit the action of insulin. Given the predominance of tyrosine kinases in tumorigenesis, it might be anticipated that PTP1B inhibition favors cancer growth, since reduced PTP1B expression was found in hepatocellular carcinomas [85]. Nonetheless, in different cancers its effects remain context dependent, since PTP1B expression driven by androgen receptor signaling, enhanced the progression of prostate cancer [86]. PTP1B expression also plays an important role in T2DM and has been found to be elevated in insulin target tissues of patients. PTP1B can directly interact with the insulin receptor [87] and insulin receptor substrate 1 (IRS-1) [88], dephosphorylating the tyrosine residues in the activation loops of these molecules to down-regulate insulin signaling, thereby contributing to an insulin resistance phenotype in T2DM patients. Transgenic *PTP1B* null mice were healthy, resisted obesity when fed with a high-fat diet and did not evolve diabetes. Restorative effects were observed by treating diabetic mice with PTP1B antisense oligonucleotides [89]. PTP1B is considered an attractive therapeutic target and among the small molecules investigated, antibodies that stabilize the oxidized conformation of PTP1B have been shown to inactivate it [90]. Favoring prospects for preventative astronaut treatment, normal “choice” metabolic responses may present therapeutic targets of more consistent behavior than heterogeneous oncogenically “chained” pathways.

One adaptation to cellular stress observed in tumors is autophagy or “self-eating” of proteins and cell organelles. It is initiated when the autophagosome, a double-membrane vesicle, engulfs cytoplasmic organelles, including mitochondria, before fusion with lysosomes that digest the vesicle’s contents. Excessive accumulation of autophagosomes can lead to cell death by apoptosis. This complex process has both tumor-suppressing and tumor-promoting activities depending on whether it is predominantly occurring in epithelial cancer cells or in stromal cells within the tumor niche [91]. Cells undergoing autophagy have fewer mitochondria and shift their metabolism towards glycolysis, producing high-energy mitochondrial fuels e.g., l-lactate, ketone bodies, glutamine and free fatty acids. Neighboring cells may consume these catabolites to fuel mitochondrial OXPHOS. Notably, p53 serves as a key component of the cellular stress response. Among its many activities, nuclear p53 can *trans*-activate numerous autophagy inducers, such as the damage-regulated autophagy modulator (DRAM), a target for silencing by hyper-methylation at its CpG islands in several tumor types [92]. Autophagy is also central to muscle glucose homeostasis in response to exercise. As shown by mice with engineered mutations affecting phosphorylation sites in the mitochondrial anti-apoptotic and anti-autophagy protein BCL2. Exercise-induced autophagy was shown to be dependent on BCL2 activity, and mutant mice defective in autophagy showed impairment of many key metabolic events, including impaired muscle glucose uptake, GLUT4 plasma membrane localization and AMPK activation [93].

Growing awareness of the oncogenic role of epigenetic alterations in genomic DNA encoding metabolically influential proteins led to recognition that fructose-1,6-biphosphatase-1 (FBP1), an antagonist of glycolysis in many cell types, was epigenetically down-regulated by methylation of its promoter sequence, mediated by NF-κB downstream of oncogenic Ras signaling. The incidence of this event sufficed for *FBP1* promoter methylation to be considered a new biomarker for predisposition to gastric cancer [94], implying that an epigenetic mechanism could also account for the Warburg effect [95]. T2DM is associated with low-grade systemic inflammation, and increased NF-κB DNA binding activity was found to be one of the characteristics of muscle tissue from T2DM patients [96], in agreement with suggestions that the NF-κB pathway may represent a therapeutic target for insulin resistance [97]. Comprehensive DNA methylation profiling in pancreatic islets of T2DM patients versus non-diabetic donors revealed aberrantly methylated disease-specific genes that are impacting pathways implicated in pancreatic β-cell survival and function. Further studies would be required to confirm to what extent this reflects causal or consequential changes [98]. Epigenetic DNA methylation is very relevant for space travel, since an irradiation response in brain tissue and inhibition of DNA methylation can be an effective neuroprotective strategy to avoid radiation-induced cognitive deficits [99]. 

A further level of molecular regulation thought to influence 74–92% of all protein-encoding mRNAs involves microRNA (miRNA) molecules that bind to the 3′ untranslated region of their target mRNA through imperfect pair bonding and effect their down-regulation. Actively involved in tumorigenesis, they can serve as oncogenes or tumor suppressor genes, depending on the context [100] and are subject to regulation by oncogenes also. Pertinently, miRNAs are implicated directly in enhancing glutamine metabolism and the Warburg effect in cancer cells [101] with several miRNAs implicated in T2DM [102]. As a specific example, miR-143 down-regulation, found in a number of tumors, can up-regulate *hexokinase 2 (HK2)* expression to promote a shift towards aerobic glycolysis in cancer cells [103]. A Warburg effect and hyperplastic metabolism via miR-143 down-regulation and *HK2* overexpression characterized esophageal squamous cell carcinomas induced by a dietary zinc deficiency [104]. It would be premature to suggest this fully defines the outcome of miR-143 expression levels, since it was up-regulated in the liver of T2DM obese mouse models, where it also impaired glucose metabolism, but through induction of insulin resistance via miR-143-ORP8-dependent inhibition of insulin-stimulated AKT activation [105]. Linking molecular metabolic mediators to dietary zinc is consistent with observations that metal elements can facilitate tissue repair processes both pre-emptively and after recovery from otherwise lethal radiation injury [106] emphasizing the importance of nutrition for protection against radiation exposure, including radiotherapy and space flight [107].

### 5.3. Lessons for T2DM from a Warburg Effect by Chance Whilst Ageing

The diabetic myotube phenotypes of increased basal glucose oxidation and incomplete lipid oxidation could be reproduced by using malonate to inhibit the TCA cycle enzyme succinate dehydrogenase. This impaired TCA cycle flux in skeletal muscle may causally contribute to a diabetic phenotype. It underscored the need for a detailed understanding of structure, regulation, modification and expression of the multifunctional TCA cycle enzymes [108]. However, with regard to an age-induced Warburg effect, the functional capacity of the mitochondria was retained in cultured senescent myotubes. Cells at late passage numbers showed a reduced mitochondrial mass and decreased whole cell ATP level, yet retained full mitochondrial ATP production capacity and increased ROS production [65]. This does not contradict studies showing that an alteration in mitochondrial oxidative and ATP generating activity was important for declined muscle function in ageing, but suggests that an indirectly evolved phenotype with ageing is per se not necessarily a direct cause of dysfunction in the TCA cycle enzymes. An age-associated lower mitochondrial mass may require the remaining mitochondria to compensate with elevated energy generation, yet at the cost of increased harmful ROS production. Notably, consistent with an age-related loss of mitochondrial mass, muscle-specific mutations that accumulated with aging were found in critical human mtDNA regions near the site of mtDNA attachment to the inner mitochondrial membrane, most likely aiming to control mtDNA replication and/or copy number [109].

Diabetes usually occurs later in life, making ageing an important risk factor. Curiously, several genes that influence the Warburg effect probably have a role in longevity. For example, sirtuins (SIRT1-7), a member of the highly conserved protein family with seven transmembrane domains, can extend the lifespan of model organisms. Targets for SIRT1 mediated deacetylation include a broad range of transcription factors and co-activators governing many central metabolic pathways, and several different models of *SIRT1* over-expression in transgenic mice were shown to prevent diabetes [110]. Notably, human fibroblasts with inactivated SIRT2 showed Warburg effect-like decreased OXPHOS and increased glycolysis [111]. SIRT3, like SIRT4 and SIRT5, resides within mitochondria and deacetylates and regulates many mitochondrial proteins. Compounding its metabolic significance, SIRT1 can deacetylate SIRT3 which becomes hyper-acetylated in aged and obese mice [112].

Genetic polymorphisms in the *SIRT3* gene promoter can influence levels of gene expression. Polymorphisms in the *PTP1B* gene have also been associated with improved health in old age. Direct comparison of relatively young (3 month) and aged (16 month) mice with a wild-type or *PTP1B*^−/−^ genetic background revealed a significant difference. Mice with *PTP1B* deficiency were protected from the development of peripheral insulin resistance, adiposity, hyperinsulinemia and islet hyperplasia found in the wild type mice at 16 months of age, indicating that this enzyme had a critical role in age-dependent onset of the murine T2DM phenotype [113]. Notably, the increase in mRNA, protein levels and PTP1B activity with obesity was confined to the liver and muscle of 16-month old wild-type mice indicating a tissue-specific response. Though upstream events elevating *PTP1B* activity were not fully defined, the transcriptional activators NF-κB, and p53 were co-elevated in the liver and muscle tissue of the 16-month old wild-type mice. p53 is known to interact with several longevity pathways, including activation of AMPK and repression of the insulin IGF1 and mTOR pathways. How tissue specific discriminatory effects may arise is far from clear, but low levels of antioxidant enzymes such as glutathione peroxidase in pancreatic β-cells compared to other tissues, may explain the increased susceptibility to oxidative stress-mediated tissue damage [114]. Appreciating an important interrelationship between p53 and mitochondria and telomeres is certainly likely to be helpful to resolve key mechanisms influencing oxidative stress metabolism that are altered by ageing [115]. 

Ageing is associated with a reduced muscle mass and strength, that can be influenced by exercise. Resistance training was shown to increase muscle strength and function even in older adults, reducing biomarkers of oxidative stress with improved mitochondrial function. Notably, sarcopenia patients, exhibited mitochondrial DNA mutations and deletions in mature myocytes, but not in their muscle satellite cells. Post-injury regeneration of skeletal muscle following strenuous exercise involved activation, proliferation and differentiation of the resident satellite stem cells and endothelial precursor cells. Tunneling nanotubes that connect myogenic skeletal muscle satellite cells to muscle fibers may serve as conduits for intracellular material, including mitochondria [116]. The concept of mtDNA shifting suggests that resistance-exercise training is leading to a replacement of defective mitochondria bearing DNA mutation and deletions with healthier mitochondria from satellite cells [117]. Though not entirely clear whether this reflected mitochondrial DNA transfer from satellite cells, or regeneration of new fibers from satellite cells with non-deleted mtDNA, recent evidence confirms that RNA-mediated improvement in mitochondrial activity could mitigate oxidative stress to drive enhanced regeneration of injured muscle [118]. In addition, bone marrow derived stromal cells can also repair tissue injury through the transfer of mitochondria via nanotubes and microvesicles [119]. Therefore, intercellular interaction is likely involved in controlling the Warburg effect, and mitochondrial transfer may be an important process in many diseases. Given that mitochondria regulate cytoplasmic radiation induced genotoxic damage [120] intercellular tunneling nanotube connections may have a role in the design of countermeasures against therapeutic and environmental radiation exposure.

## 6. Not Forgetting the Reverse and Inverse Warburg Hypothesis

In multicellular tumor microenvironment models, cancer cell acquired mutations that can directly or indirectly favor ROS generation [121,122]. The “reverse Warburg” hypothesis proposes that in response to increased local hydrogen peroxide levels, it is the adjacent cancer-associated fibroblasts rather than cancer cells themselves [123] that undergo most of the aerobic glycolysis [124]. In turn, ATP produced by stromal cells may help cross-feed cancer cells to compensate mutations and maintain mitochondrial activity. This would allow cancer cells to proceed with an active TCA cycle and with oxidative phosphorylation pathways to meet the enhanced ATP and anabolic demand of proliferating tumor cells.

Helping to support this scenario, cancer cells express cytoprotective genes that sustain anabolic metabolism despite the oxidative pressure in their microenvironment. Pivotal for both enhancing metabolism and protection, the transcription factor Nuclear erythroid factor 2-like (Nrf2), constitutively active in many cancer cell types, can redirect glucose and glutamine into anabolic pathways, including purine nucleotide generation in the presence of PI3K-Akt signaling [125]. The broad repertoire of transcriptional targets directed by Nrf2 include at least six metabolic regulator genes involved in the PPP and NADPH production pathways, plus numerous anti-oxidant enzymes and cytoprotective factors [126]. Somatic mutations inactivating Kelch-like ECH-associated protein 1 (Keap1), the canonical inhibitor of Nrf2, are relatively frequent, preventing Nrf2-Keap1 interaction in the cytoplasm and promoting proteasomal degradation of Nrf2. Mutations in *Nrf2* itself are frequently disrupting discs-large (DLG) or glutamate-threonine-glycine-glutamate (ETGE) motifs associated with low or high affinity Keap1 interactions, thus favoring constitutive activation of Nrf2 [127]. One may regard Nrf2 as a nefarious complement to oxidative and hypoxic environments that facilitates tumor growth [128]. However, in normal cell metabolism contexts, the repressive action of Nrf2 on ATP citrate lyase, Acc1 and Fasn, can suppress *de novo* lipogenesis which is required for tumor growth. Accordingly, Nrf2 has a highly context-dependent role, permissive for already initiated cancer growth yet also beneficial for cancer prevention, since absence of Nrf2 also reduces DNA repair in normal cells [129], increasing cell susceptibility to carcinogenic agents [130]. Astronauts resident in the international space station for six months showed ROS alterations thought to reflect reduced mitochondrial synthesis and limited expression of genes including *Nrf2*, highlighting a potentially increased cancer risk in astronauts [131].

Besides cancer and diabetes, the Warburg effect influences numerous morbidities. Although it improves host responses to pathogens through T cell activation [132], the Warburg effect is generally associated with progression of chronic disease. It is implicated in multiple sclerosis, pulmonary hypertension and idiopathic pulmonary fibrosis, cardiac hypertrophy, atherosclerosis, polycystic kidney disease and Alzheimer’s disease (AD) [133]. Notably, for the latter, a hypothesis termed “inverse Warburg” effect has been proposed. Distinct from the “reverse Warburg” hypothesis of tumorigenesis, it nevertheless involves a reciprocal metabolic coupling between adjacent cell types [134], namely neurons and microglia. Proposed by Demetrius et al., the bioenergetics-based inverse Warburg model for sporadic forms of AD implicates energy generation and age as critical elements in the origin of this neurodegenerative disease. As a first step, age-induced chance inefficiency in the mitochondrial activity [135], such as that mediated by DNA protein kinase within certain neurons [136] causes a compensatory ramped up-regulation of oxidative phosphorylation to maintain cell viability. In effect, this is a reversal of Warburg effect pathways that replace oxidative phosphorylation with aerobic glycolysis. Subsequently, a cascade of events evokes a selective advantage for neurons with mild mitochondrial impairment and increased OXPHOS activity over healthy neurons. Neurons adopt this option due to low expression levels of *PFKFB3* encoding the 6-phosphofructo-2-kinase/fructose-2,6-biphosphatase 3 enzyme that is critical for the regulation of glycolysis [137]. Thus, neurons have a limited capacity to up-regulate glycolysis for compensating the increased energy demands. In contrast, microglial astrocytes express high levels of PFKFB3 and also show deficiencies in pyruvate dehydrogenase activity and elements for shuttling cytosolic NADH within mitochondria for pyruvate oxidation instead of lactate formation. Accordingly, neurons are essentially oxidative cells, whereas astrocytes have high glycolytic capacity [138] and use this alternative metabolic route to generate lactate that can fuel adjacent neurons. Defective neurons with excessive OXPHOS activity produce ROS that promote pathological ageing, with abnormal cell cycle entry and eventual neuronal loss. At the same time, production of lactate or ketone bodies can be considered a compensatory response in adjacent microglia to meet the high energy demands of defective neurons. The proposal that intercellular Warburg effect-mediated interactions may modulate disease progression [139] can help account for idiosyncrasies surrounding a metabolic amyloid cascade hypothesis, whereby aggregation of neurite plaques, with consequential disruption of synaptic connections, underlies the death of neurons and the overwhelming forgetfulness of dementia [140].

## 7. New Light on Redox Metabolism Pathways in Extreme Conditions

Historically, numerous medical advances trace origin to causal relationships from extreme examples providing unique peculiarities that highlighted key functional mediators, e.g., the occupational hazard of early 20th century watch dial painters, who pointed brushes in their mouths and contracted malignancies from radioluminescent radium poisoning [141]. A current extreme occupational endeavor inevitably associated with numerous metabolic risks is human spaceflight, with astronauts being subjected to unique microgravity and ionizing radiation conditions [142].

Weightlessness from microgravity drives dramatic physiological changes, including an altered cardiovascular system and circulation, immunosuppression and changes to calcium, sodium and bone metabolism [143]. Nonetheless, it is notable that an additional higher cardiovascular disease mortality risk can be found in Apollo lunar astronauts, the only humans to have travelled beyond Earth’s radiation-shielding magnetosphere, versus astronauts that have only flown in low Earth orbit. Advances in systems biology provide an improved interpretation of the molecular signatures and networks altered in human cells by microgravity and ionizing radiation-induced oxidative stress [144], highlighting a close association among oxidative phosphorylation/respiratory electron transport, ubiquitin-proteasome system and neurodegenerative conditions (e.g., Alzheimer’s disease) across multiple datasets. Mice genetically modelling AD exposed to 56Fe ion particle radiation showed an accelerated age-associated accumulation of amyloid beta (Aβ) plaques and increased cognitive impairment [145]. This concurred with later studies showing neuronal network fragility to the combination of simulated microgravity and chronic exposure to radiation [146]. Extending repercussions beyond generation of ROS, immune dysfunction induced by spaceflight has been closely associated with metabolic change in fatty acid oxidation and decreased glycolysis-related pathways [147]. 

Fortunately, experimental methods applicable to the analysis of the Warburg effect and mitochondria are advancing. Refined isolation of mitochondria and measurement of mitochondrial membrane potential and metabolites has accelerated screening of potential new drugs that shift energy metabolism from mitochondrial OXPHOS to aerobic glycolysis [148]. Powerful proteomic approaches allow broad scale analysis of metabolite profiles, highlighting associations between amino acid metabolites and the risk of developing diabetes [149]. A comprehensive metabolic signature has been obtained for defining the Warburg effect in situations such as pancreatic cancer [150]. Spectrophotometric protocols allow simple and reliable assessment of respiratory chain function to be applied to minute quantities of muscle tissue, cultured cells and isolated mitochondria [151]. 

To date, the current repertoire of nutritional supplements has done little to provide true clinical benefit [152]. However, improved, more sophisticated transgenic mouse models will allow better discrimination between cause and effect when exploring metabolic disorders. Despite only partial mimicry, simulated microgravity ground experiments can suffice to potentiate the effect of ROS [153] shown to exacerbate the effects of heavy ion radiation in human B lymphoblasts [154]. Dedicated heavy-ion radiobiology research centers, e.g., the NASA Space radiation laboratory at Brookhaven, are pivotal for improving space-relevant radiobiology knowledge and exploration of how heavy-ion species might be exploited for focused anti-cancer therapy [155]. Combining heavy-ion irradiation from a carbon ion combined with a clinostat to simulate microgravity allowed exploration of synchronous effects pertinent to space radiation research [156]. Additional technological advances include laser-plasma-accelerators representing advantageous tools for accurately reproducing broadband radiation particle flux similar to conditions in space [157]. Prospective research adopting Extreme Light Infrastructure-Nuclear Physics (ELI-NP) at the Center for Advanced Laster Technologies (CETAL) facility, will provide two very high intensity 10 PW lasers and a very intense (10^13^ γ/s) brilliant γ beam with which to explore appropriate shielding methods [158] for deep space flights and for developing relevant radiobiology investigations.

## 8. Conclusions

The Warburg effect hypothesis, originated from findings in cancer cells, has proved helpful to better understand key mechanisms underlying a broad range of metabolic diseases. Certainly, the situation is extremely complex, and many examples of anaerobic glycolysis co-existing with normal TCA function and OXPHOS pathways suggest a dynamically regulated shared metabolic balance rather than strict adoption of one mode of respiration at the expense of the other. Biomarkers for oxidative stress assessed in broader population studies of environmental metabolic stressors may have a direct causal role and represent indirect indicators of questionable therapeutic value or serve as good intervention targets [159]. Assumptions that measurements made ex vivo apply to in vivo microenvironments need confirmation [160] because, in a heterogeneous tissue, clusters of abnormality become risk factors in their own right. The impact of an event such as mtDNA mutation with regard to disease conditions may vary between different cell types, different tissues and even different human populations [161]. Molecules exerting significant phenotypic effects in more than one disease state are being discovered, and a broader network view can improve our interpretation of how metabolic mechanisms are coordinated. Applying a choice, chain, chance perspective to integrate molecular metabolic pathways influenced by the Warburg effect in developmental biology, cancer and type 2 diabetes mellitus can provide unifying insights.

The scope of relevance for the Warburg effect has extended well beyond its origins as an anomaly of cancer. It is clear that, for any long-term space mission, astronauts will need appropriate physical shielding and additional medicinal countermeasures to limit both excessive oxidative stress triggered by ionizing radiation and the associated inflammatory processes mediated by the NF-κB pathway [162]. Use of cytoprotective agents, such as Amifostine, can provide radioprotection to normal tissues by triggering a metabolic shift with induction of glycolysis and blockage of mitochondrial pyruvate usage through a Warburg effect pathway that reduces ROS production [163]. A possible therapeutic solution might be the use of Nrf2 activators, such as dimethyl fumarate and sulforaphane, for enhancing the endogenous antioxidant protection against the oxidative pressure of galactic cosmic radiation [164]. Quantitative analysis of low-molecular mass endogenous metabolites is becoming ever-more achievable with modern analytical techniques that are well-suited to complex pathologies [165]. It is anticipated that space exploration will continue to be a major stimulus for advancing our understanding of human metabolism [166] and reciprocate with unique experiments that extend our capacity to overcome even the most challenging complex human diseases and environmental pressures.
